# Drug challenge test for early symptom control in patients with coronary artery spasm: a case report

**DOI:** 10.1093/ehjcr/ytaf134

**Published:** 2025-04-08

**Authors:** Yoshiaki Kawase, Kento Kikuchi, Takuya Mizukami, Hitoshi Matsuo

**Affiliations:** Department of Cardiology, Gifu Heart Center, 4-14-4 Yabutaminami, Gifu City, Gifu 500-8384, Japan; Department of Cardiology, Gifu Heart Center, 4-14-4 Yabutaminami, Gifu City, Gifu 500-8384, Japan; Department of Cardiology, Gifu Heart Center, 4-14-4 Yabutaminami, Gifu City, Gifu 500-8384, Japan; Department of Cardiology, Gifu Heart Center, 4-14-4 Yabutaminami, Gifu City, Gifu 500-8384, Japan

**Keywords:** Coronary artery spasm, Symptom control, Prescription, Efficacy, Outpatient department, Case report

## Abstract

**Background:**

Calcium channel blockers are the first-line treatment option, followed by long-acting nitrates or nicorandil as second-line medications for patient with coronary artery spasm (CAS). However, there are cases where symptoms cannot be controlled by a combination of these drugs. The drug choice after first- and second-line treatment options is varied and challenging.

**Case summary:**

A 70-year-old woman presented to our hospital with complaints of angina at rest. The patient was diagnosed with CAS based on a positive acetylcholine provocation test result. Nitrates were intolerable due to headaches. The combination of calcium channel blocker and nicorandil was not effective in mitigating her symptoms. Four potential symptom relief drugs—trimetazidine, shigyakusan, keishibukuryogan, and denopamine—were prescribed. Each drug was administered for one week, and symptom improvement was assessed one month later. Two drugs (shigyakusan and keishibukuryogan) were effective in relieving her symptoms, but neither was satisfactory on its own. Therefore, these two drugs were combined and added on top of the calcium channel blocker and nicorandil. Her symptoms were well controlled thereafter.

**Discussion:**

The drug challenge test, which involves prescribing various types of drugs for short durations to evaluate their effects, may be an effective option for quickly controlling symptoms in patients with refractory CAS who exhibit frequent symptoms.

**Learning point**

- Drug challenging test might be an effective option to quickly control the symptoms in patients with refractory coronary artery spasm who exhibit frequent symptoms.

## Introduction

Calcium channel blockers (CCBs) are the first-line treatment option for coronary artery spasm (CAS). Standard doses of CCBs fully prevent angina attacks in 80%–90% of patients.^1,2^ Some patients need a combination of dihydropyridine and non-dihydropyridine type CCBs, or the additional use of long-acting nitrates or nicorandil as second-line medications.^3^ However, there are cases where symptoms cannot be controlled by a combination of these drugs. Choosing the appropriate drug after first- and second-line treatment options is varied and challenging.

## Summary figure

**Figure:**
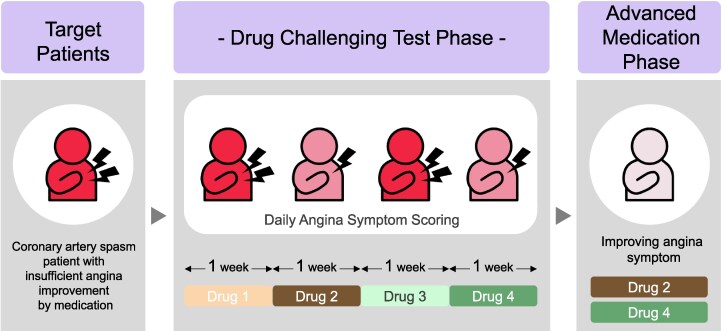
The conceptual figure of drug challenging test.

## Case presentation

A 70-year-old woman presented to our hospital with complaints of angina. Her symptoms occurred at rest or during sleep, and no symptoms were observed during daytime activity. She experiences chest pain every day, sometimes more than 10 times a day. Nitroglycerine was effective in suppressing the symptoms but was difficult to use due to headaches and the frequency of her symptoms. She has no history of smoking or other coronary risk factors. Her blood pressure was 100/82 mmHg and heart rate was 85 beats per minute without any medication. Neither a 12-lead electrocardiogram nor an echocardiogram showed abnormal findings. An invasive angiogram was planned to determine the cause of her symptoms. Vasodilatory drugs were temporarily stopped at least 48 h before the procedure. The invasive coronary functional testing to diagnose CAS was conducted in accordance with the guideline of the country.^4^

**Figure 1:**
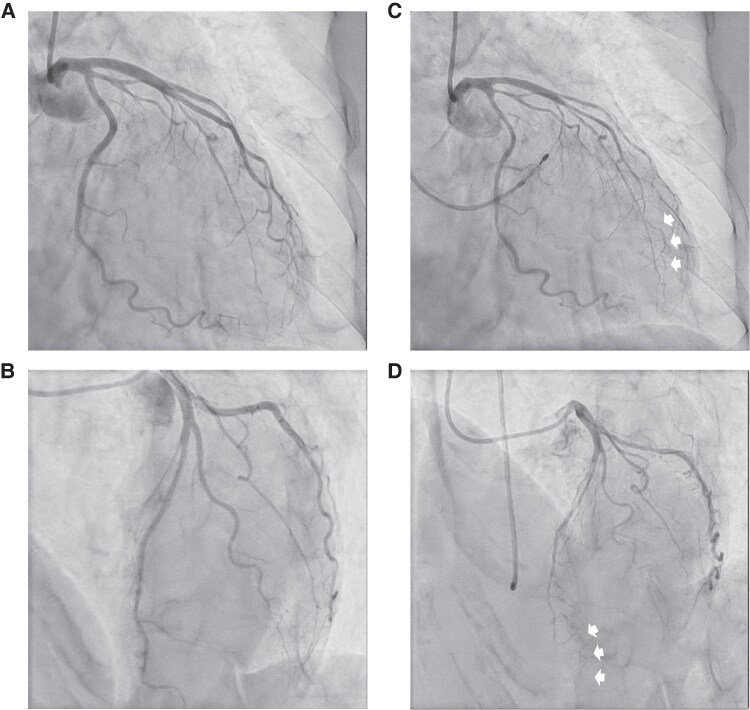
Invasive coronary angiogram. (*A* and *B*) Baseline invasive coronary angiogram of LCA, showing no organic stenosis in the left coronary artery. (*C* and *D*) Coronary angiogram during the acetylcholine provocation test. Severe spasm was provoked at distal part of the left anterior descending coronary artery (white arrows).

Briefly, after confirming the absence of significant organic stenosis in the patient’s coronary arteries, acetylcholine dissolved in 37°C saline was injected into the left (20, 50, and 100 μg) and right (20 and 50 μg) coronary arteries over 20 s. In this case, the baseline angiogram showed no significant organic stenosis in her coronary arteries. During the acetylcholine provocation test, a dose of 10 μg (midway through the 20 μg injection) into the left coronary artery induced a diffuse, severe vasospasm in the distal part of the left anterior descending coronary artery (90% diffuse vasoconstriction across two or more contiguous segments), reproducing her daily symptoms and meeting the definition of CAS. (*Figure 1A–D*)

**Figure 2:**
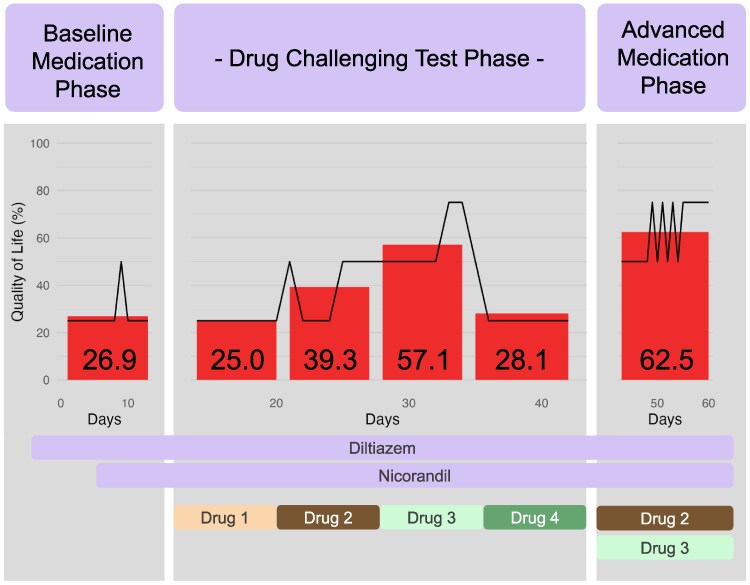
The transition of Quality-of-Life score with each drug. Longitudinal axis: Quality-of-Life score (%). Horizontal axis: days after treatment initiation. Numbers shown within the bars indicate the average Quality-of-Life score (%) for each drug over a week. Drug 1: trimetazidine (25.0%), drug 2: shigyakusan (39.3%), drug 3: keishibukuryogan (57.1%), drug 4: denopamine (28.1%), combination of drugs 2 and 3 (62.5%).

The patient was diagnosed with CAS, and control of her symptoms was attempted at the outpatient department. Nitrates were intolerable due to headaches. The combination of dihydropyridine and non-dihydropyridine CCBs was not ideal considering her low blood pressure at baseline. The combination of non-dihydropyridine CCB and nicorandil was not effective in mitigating her symptoms. Four potential symptom relief drugs—trimetazidine, shigyakusan, keishibukuryogan, and denopamine—were prescribed.^4,5^ The latter three are exclusively highlighted in the guidelines of the country.^4^ Each drug was administered for one week, and we assessed symptom improvement one month later. Two drugs (shigyakusan and keishibukuryogan) were effective in relieving her symptoms, but neither was satisfactory on its own. Therefore, these two drugs were combined and added on top of the CCB and nicorandil. Her symptom was well controlled thereafter (*Figure 2*).

## Discussion

The present case involved a patient with refractory CAS who did not respond to conventional first- and second-line medications. It is important to quickly control the symptoms of CAS patients, not only for their well-being but also to avoid the effects of disease activity related to seasonal changes.^6^ If it takes several months or more to manage the symptoms, they might be relieved by other factors such as changes in seasonal disease activity.

However, the protocol for using drugs for CAS is not clear after trials of CCBs, long-acting nitrates, and nicorandil. There are case reports showing effective drugs for refractory CAS when first- and second-line treatments fail, but their selection is varied,^7–10^ and most do not have concrete evidence.^11,12^ The trial of these drugs in practice is usually conducted in the outpatient department, and weekly visits might place a burden on both patients and medical staff. Therefore, we implemented a ‘drug challenge test’, which involved prescribing four types of potential symptom relief drugs, each for one week, and checking for symptom improvement one month later. The Quality-of-Life score was reported by the patient through an internet questionnaire system daily. By utilizing this system, we could evaluate the average Quality-of-Life score for each drug over a week and identify one or more promising drugs for symptom control.

In this case report, we utilized a combination of four drugs, some of which are only available in Japan. Alternative therapies commonly considered for similar regimens include ranolazine, ivabradine, zibotentan, and trimetazidine.^5^ These drugs function through distinct mechanisms: ranolazine inhibits late sodium currents, reducing myocardial ischaemia; ivabradine selectively inhibits the If current in the sinoatrial node to lower heart rate and myocardial oxygen demand; zibotentan, a selective endothelin A receptor antagonist, alleviates coronary vasoconstriction and improves endothelial function; and trimetazidine enhances myocardial energy efficiency by shifting energy metabolism from fatty acid oxidation to glucose utilization.

The specific combination of drugs can be tailored depending on the clinical context and drug availability in each region. Additionally, during follow-up, the regimen can be adjusted based on the intervals between consultations. For example, if follow-up visits are scheduled at four-week intervals, up to four drugs can be tested sequentially, as demonstrated in this case. However, if the interval is three or five weeks, three or five drugs may be trialled, respectively. It is important to note that increasing the number of drugs may reduce the accuracy of patient recall, potentially impacting the reliability of symptom evaluation and overall assessment without an internet-based questionnaire system like the one used in our case report.

There are several limitations to this method. First, patients should experience symptoms almost daily to accurately assess the effect of each drug within one week. Second, patients must have sufficient cognitive ability to recall the effects of each drug and report them through an internet questionnaire system. Third, some drugs only begin to show effects after one or two weeks of administration; therefore, these drugs should not be evaluated using this method.

Finally, we did not perform a test for microvascular function in this patient. Coronary and peripheral microvascular dysfunction are known to play a role in patients with CAS.^4,5,13^ The patient’s refractory response to drugs may reflect the involvement of microvascular dysfunction.

## Conclusion

A drug challenge test may be an effective option for quickly controlling symptoms in patients with refractory CAS who experience frequent symptoms.

## Data Availability

The data underlying this article will be shared on reasonable request to the corresponding author.

## Lead author biography

Yoshiaki Kawase is an interventional cardiologist who graduated from Gifu University School of Medicine, Gifu, Japan, in 1998. Since 2010, he has been serving as a co-director of the catheterization laboratory at Gifu Heart Center in Gifu, Japan. Memberships: The Japanese Society of Internal Medicine, The Japanese Circulation Society, and The Japanese Association of Cardiovascular Intervention and Therapeutics.

## Notes

**Consent:** The authors confirm that written consent has been obtained from the patient for the submission and publication of this case report, including images and associated text, in accordance with COPE guidance.

**Funding:** There is no funding regarding this case report.
